# LR Hunting: A Random Forest Based Cell–Cell Interaction Discovery Method for Single-Cell Gene Expression Data

**DOI:** 10.3389/fgene.2021.708835

**Published:** 2021-08-20

**Authors:** Min Lu, Yifan Sha, Tiago C. Silva, Antonio Colaprico, Xiaodian Sun, Yuguang Ban, Lily Wang, Brian D. Lehmann, X. Steven Chen

**Affiliations:** ^1^Department of Public Health Sciences, Miller School of Medicine, University of Miami, Miami, FL, United States; ^2^Sylvester Comprehensive Cancer Center, Miller School of Medicine, University of Miami, Miami, FL, United States; ^3^Dr. John T. Macdonald Foundation Department of Human Genetics, Miller School of Medicine, University of Miami, Miami, FL, United States; ^4^John P. Hussman Institute for Human Genomics, Miller School of Medicine, University of Miami, Miami, FL, United States; ^5^Department of Medicine, Vanderbilt University Medical Center, Nashville, TN, United States; ^6^Vanderbilt-Ingram Cancer Center, Vanderbilt University Medical Center, Nashville, TN, United States

**Keywords:** random forests, ligand-receptor interaction, cell–cell interaction, cell–cell communications, single-cell RNA-seq

## Abstract

Cell–cell interactions (CCIs) and cell–cell communication (CCC) are critical for maintaining complex biological systems. The availability of single-cell RNA sequencing (scRNA-seq) data opens new avenues for deciphering CCIs and CCCs through identifying ligand-receptor (LR) gene interactions between cells. However, most methods were developed to examine the LR interactions of individual pairs of genes. Here, we propose a novel approach named LR hunting which first uses random forests (RFs)-based data imputation technique to link the data between different cell types. To guarantee the robustness of the data imputation procedure, we repeat the computation procedures multiple times to generate aggregated imputed minimal depth index (IMDI). Next, we identify significant LR interactions among all combinations of LR pairs simultaneously using unsupervised RFs. We demonstrated LR hunting can recover biological meaningful CCIs using a mouse cellular indexing of transcriptomes and epitopes by sequencing (CITE-seq) dataset and a triple-negative breast cancer scRNA-seq dataset.

## Introduction

In recent years, single-cell RNA sequencing (scRNA-seq) has been widely applied to measure gene expression at single-cell resolution, and has become a powerful tool to detect common and rare cell subpopulations, construct cell lineage and pseudotime, and identify spatial gene expression pattern, etc. While there still are many open problems and challenges remaining, scRNA-seq data analysis can be further expanded and developed to fully utilized the data for better understanding the cell heterogeneity and gene expression stochasticity (Lahnemann et al., 2020).

Cell–cell interactions (CCIs) and cell–cell communication (CCC) are crucial for cell development, tissue homeostasis, and immune interactions in multicellular organisms (Armingol et al., 2021). In the case of cancer, tumor cells can reprogram their microenvironment to turn neutral or anti-tumor cells into tumor supportive elements (Hanahan and Weinberg, 2011; Junttila and de Sauvage, 2013), partly through secreted ligand and cell surface receptor physical interactions (Ramilowski et al., 2015). The availability of scRNA-seq data provides the great opportunities to decipher the CCIs and CCC through ligand-receptor (LR) gene expressions (Shao et al., 2020; Liu et al., 2021). Several analysis tools have been developed to infer CCC by modeling the LR co-expression data including Spearman correlation between LRs (Zhou et al., 2017; Cohen et al., 2018), product-based score from gene expression of LR pair (Kumar et al., 2018; Cabello-Aguilar et al., 2020; Hu et al., 2021), differential gene combinations (Tyler et al., 2019; Cillo et al., 2020), gene expression permutation test (Efremova et al., 2020; Dries et al., 2021; Noel et al., 2021).

Most available CCC analysis methods quantify each LR pair separately. However, biologically CCIs and CCC happen in much more complicated scenarios. In particular, the multiple ligands can compete with each other for binding on the same receptor. Therefore, the LR relationships may not be one-to-one, but would be many-to-one or many-to-many instead. To better capture the complex relationships between LR interactions, here we propose a new multivariate CCC analysis approach based on random forests (RFs), which incorporates the correlations and interactions among intercellular networks to rank and prioritize the LR interactions.

## Method

### LR Hunting Modeling

We present a machine learning framework for LR interaction discovery, which can be used to analyze any curated LR database such as FANTOM5 (Ramilowski et al., 2015), IUPHAR (Harding et al., 2018), DLRP (Graeber and Eisenberg, 2001), or CellPhoneDB (Efremova et al., 2020).

#### Gene Expression Data Imputation

**FIGURE 1:**
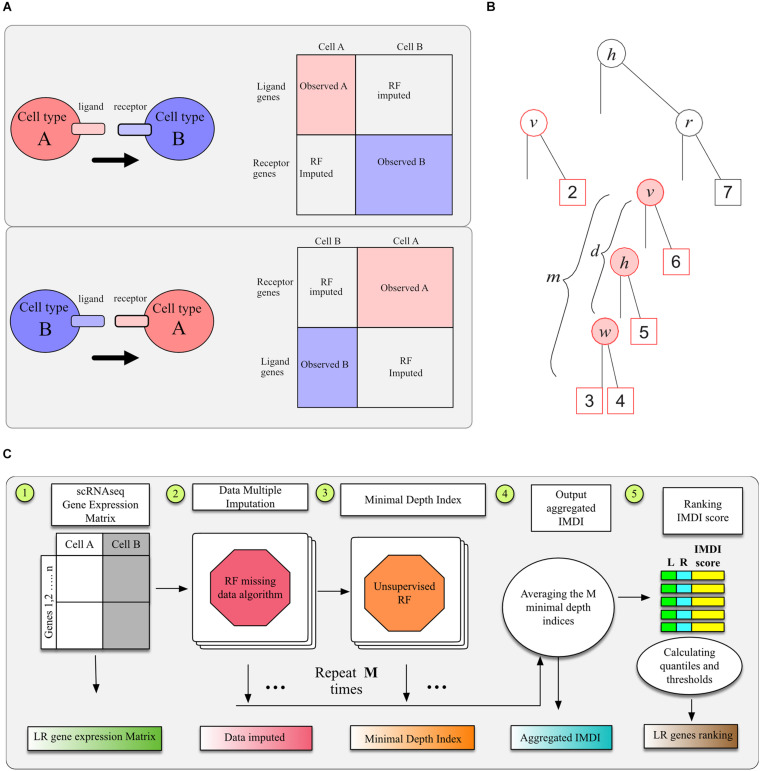
Illustration of RF methods. **(A)** Data sheet and imputation illustration. **(B)** The minimal depth of *w* in a maximal *v*-subtree. Letters in parent nodes identify the variable used to split the node. There are two maximal *v*-subtrees, marked in red. The maximal *v*-subtree on the left side is with terminal nodes 1 and 2; that on the right side is with terminal nodes 3, 4, 5, and 6. The minimal depth of *w* in the second maximal *v*-subtree is the depth of *w* (*d* = 2 marked with pink background) normalized by the subtree depth (*m* = 3), which is *d/m* = 2/3. **(C)** Model workflow for LR hunting.

To identify LR interactions between two cell types using LR hunting analysis, we need to build the complete pseudo gene expression data matrix since ligand genes and receptor genes are from different cell types in the “interaction space” (Figure 1A). We assume that the gene expressions between two cell types follow a multivariate distribution *p* so that all the gene expression can be observed or imputed in the same framework. Formally, denote *X*^(*A*)^ as an *n*_*A*_×*p*_*A*_ matrix that records ligands gene expression for cell type *A* and let *X*^(*B*)^ be an *n*_*B*_×*p*_*B*_ matrix that records receptor gene expressions for cell type *B*. Our goal is to obtain an (*n*_*A*_ + *n*_*B*_)×(*p*_*A*_ + *p*_*B*_) matrix *x*∼*p* so that gene associations or interactions between cell types *A* and *B* can be computed using multivariate approaches. If we are interested in the interactions between ligand genes from cell type *B* and receptor genes from cell type *A*, imputation procedure can be performed similarly as we illustrated in Figure 1A.

To this end, we applied a machine learning model, the RF missing data imputation algorithm developed by Tang and Ishwaran (2017), which was shown to be as an efficient multivariate imputation approach for high-dimensional genomic data. The RF technology is related to recursive partitioning and regression tree analyses. A single tree is inherently unstable, hence a forest of trees is “grown” from bootstrap samples of the original dataset, where an average of 37% of the data will not be sampled, referred as out-of-bag (OOB) data. The forest permits an ensemble average to be calculated across the individual trees (Breiman, 2001). We adopted the unsupervised splitting rule, where a random set of *q* variables, say *X*_1_,…,*X*_*q*_, is selected to be the multivariate pseudo-predictors. Let *s* be a proposed split for a pseudo-predictor *X_i* that splits the node *t* into left and right daughter nodes *t*_*L*_ = {*X*_*i*_≤*s*} and *t*_*R*_ = {*X*_*i*_ > *s*}. For continuous variables, the best split is to minimize the split-statistic

$$D_{q}\,\left(s,t\right)=\sum_{k=1}^{q}\left\{\sum_{j\in t_{L}}{\left(X_{j,k}-{\bar{X}}_{t_{L_{k}}}\right)}^{2}+\sum_{j\in t_{R}}{\left(X_{j,k}-{\bar{X}}_{t_{R_{k}}}\right)}^{2}\right\},$$

Where, ${\bar{X}}_{t_{L_{k}}}$ and ${\bar{X}}_{t_{R_{k}}}$ are the sample means of the *k*-th pseudo response coordinate in the left and right daughter nodes. The imputation utilized the above multivariate unsupervised splitting rule for each tree where missing values are first discarded. After the forest is grown, missing data are imputed using OOB non-missing terminal node data.

#### Unsupervised Random Forests Minimal Depth Index

In order to detect LR interactions in a multivariate fashion, we adopted the unsupervised RF approach to analyze the imputed data (Shi and Horvath, 2006; Mantero and Ishwaran, 2021). RF is a modern machine learning technique that permits exploration of complex, non-linear interrelationships (Breiman, 2001; Chen and Ishwaran, 2012). Its extension to an unsupervised algorithm composes two steps. The first step involves generating a synthetic dataset by drawing an equal number of observations from the corresponding predictor variable marginal distributions. The second step utilizes a multivariate RF to predict the synthetic features so that multivariate impurity splitting is able to applied in a supervised fashion.

Although the unsupervised RF can be used to cluster cells, we are more interested in selecting genes that interact with each other. We applied the minimal depth index to evaluate LR interactions in RF models (Ishwaran et al., 2010, 2011; Chen and Ishwaran, 2013). With forests, one often observes informative variables tending to split close to the root node, where the closeness is measured by minimal depth. When considering a maximal *v*-subtree (Ishwaran, 2007), we could use the minimal depth of variable *w* to quantify the interaction between variables *v* and *w*. To illustrate this, we denote *T* as a random tree and define *T_v* a *v*–subtree in *T* for any variable *v* if the root node of *T_v* is split using *v*. We call *T_v* a maximal *v*–subtree if *T_v* is not a subtree of a larger *v*–subtree and define the minimal depth statistics of *v*, denoted by *D_v*, as the distance from the root node of *T* to the root of the closest maximal *v*-subtree. For example, there are two maximal *v*-subtrees in Figure 1B, marked in red. The maximal *v*-subtree on the left side is with terminal nodes 1 and 2; that on the right side is with terminal nodes 3, 4, 5, and 6.

We denote the maximal *w*–subtree in *T_v* as *T*_v,w_ is *w* is used for the daughter nodes of *T_v* and *T*_v,w_ is not a subtree of a larger *w*–subtree in *T_v*. The minimal depth from *v* to *w* in *T_v* equals to the distance from the root node of *T_v* to the root of the closest maximal *w*–subtree *T*_v,w_, which is denoted as *D*_*v,w*_. Let *m* be the depth of subtree *T*_v,w_ and let *l* be the depth of the entire tree *T.* Assuming *v* and *w* are weak variables and independent with each other, we have

(1) $$\mathbb{P}\left(D_{v,w}=d\right)=\sum_{m=d}^{l}\mathbb{P}\left(D_{v}=l-m\right)\mathbb{P}\left(D_{w}=l-m+d\right).$$

It was deducted that *ℙ*(*D_v_* = *s*) = (1−1/*p*)^2*s*−1^ [1−(1−1/*p*)^2*s*^], which makes Equation 1 a complicated function of *d* and *l* (Ishwaran, 2007). From this, we can normalize *D*_*v,w*_ using the cumulative distribution function *ℙ*(*D*_*v*,*w*_≤*d*) to evaluate LR interactions. A simpler way to normalize *D*_*v,w*_ is *d*/*m*, which gives similar ranks for interactions according to empirical results.

As illustrated by Figure 1B, the interaction between variables *v* and *w* is marked with pink background: when these two variables interact with each other, we expect this depth to be smaller and this close split pattern to be repeated frequently among different trees. A single tree can be used to calculate multiple minimal depths of variables in multiple maximal subtrees, such as variables *h* and *v* in Figure 1B, where the maximal *h*-subtree is the entire tree. The minimal depth *D*_*v*,*w*_ = *d*is normalized by the depth of the corresponding subtree as *d*/*m* and normalized values from different maximal *v*-subtrees are averaged across the entire forest. We could detect variable interactions in a multivariate way adopting this imputed minimal depth index (IMDI), which averages the normalized *D*_*v,w*_ and *D*_*v,w*_. This normalized index ranges from 0 to 1 and smaller values indicate stronger interaction effects.

To enable the imputed dataset robustly represents the underlining distribution *p*, we adopt the idea of multiple imputation, a general approach to allow for the uncertainty about the missing data by creating several different plausible imputed datasets and combining results obtained from each imputed dataset (Harel and Zhou, 2007; Carpenter and Kenward, 2014). Specifically, we generate imputed dataset **X**_*m*_,*m* = 1,…,*M*, from our RF data imputation procedure described in the previous section, and use the generated IMDI, denoted by *I*_(*m*)_(*S*) to identify interaction for gene pair *S* across imputed dataset. We define the aggregated IMDI for gene pair *S* as

$$I\,\left(S\right)=\frac{1}{M}\,\sum_{m=1}^{M}I_{\left(m\right)}\,\left(S\right).$$

There are *p*_*A*_×*p*_*B*_ pair of potential interactions calculated, and we use the empirical distribution of *I*(*S*) from these pairs to determine the threshold of significant interactions. The whole procedure to calculate *I*(*S*) is illustrated in Figure 1C. We tested replication number *m* from 5, 10, 20, 50, 100, to 200 and found that the aggregated IMDI index was stable after 20 replications. We used 20 imputed datasets and aggregated those 20 IMDI for the analysis in section “Result.”

Random forest hunting was implemented in the open-source R software using the randomForestSRC. From the randomForestSRC R package, the function rfsrc was used for data imputation under default setting with 1,000 trees except we set na.action = “na.impute”; then minimal depth indices were estimated using the function find.interaction with method maxsubtree. LR hunting analysis code is available is at https://github.com/TransBioInfoLab/LRinteractions.

### Pre-processing and Normalization of scRNA-seq Dataset

Two scRNA-seq datasets were used to illustrate the LR hunting approach. The first dataset is a high-quality cellular indexing of transcriptomes and epitopes by sequencing (CITE-seq) of murine spleen containing 7,097 cells with more than 1,200 mRNA unique molecular identifiers (UMIs) (Govek et al., 2021). Another dataset is scRNA-seq data from five primary triple-negative breast cancer (TNBC) including 24,271 cells and 6,125 UMI detected per cell (Wu et al., 2020). For both datasets, the SCTransform function from the R package Seurat_3.1.0 was used for scRNA-seq data normalization before applying LR hunting algorithm. The CellAssign was applied to annotate cell type for murine spleen CITE-seq data (Zhang et al., 2019).

### scRNA Visualization

A Seruat object was created (CreateSeuratObject, min.cells = 3, min.features = 200) with the R package Seruat (version 3.2.3) (Stuart et al., 2019) from logNormalized scRNA from five TNBC tumors. For clustering, the following parameters were used: RunPCA; RunUMAP, dims = 1:30; FindNeighbors (dims = 1:30); and FindClusters. UMAP plots were generated and colored by expression levels of cell lineage markers to identify cell populations and interactions. Individual cells were plotted using previously published cell types or expression of interesting LR pairs (Wu et al., 2020).

### Circos Plot Visualization of Ligand-Receptor Interaction

To summarize interactions among cell types, individual gene pair ranks were summed across the five individual patients. LR interactions were visualized with circos plots colored by interaction strength (rank sum) and line thickness representing the frequency of interaction across the tumors. Arrows indicate direction of ligand to receptor pair between cell types. Circos plots were generated using the R package circlize (Gu et al., 2014).

## Results

### LR Hunting Recovered the Validated Cell–Cell Interactions Using scRNA-seq Data

The new digital image technologies and pipelines for multiplexed immunohistochemistry (mIHC) such as CO-Detection by indexing (CODEX) can quantify the antigens at the single-cell level to characterize tissue spatial architecture (Goltsev et al., 2018). A very recent new analysis method, spatially-resolved transcriptomics *via* epitope anchoring (STvEA), can integrate the CITE-seq data with mIHC images to achieve high-resolution of annotation for cell populations in the mIHC data to uncover the spatial transcription patterns (Govek et al., 2021). STvEA integrated CITE-seq and CODEX information to identify the LR pairs, thus the results are reliable and accurate. We applied LR hunting approach to only the scRNA-seq data from murine spleen CITE-seq data and then compared our results with those obtained using STvEA.

More specifically, we focused on three spatially colocalized cell populations including monocyte-derived macrophages, red-pulp macrophages, and neutrophils. We followed the procedures and LR annotations described in Govek et al. (2021). First, the mouse gene symbols were converted to the human ortholog symbols using the Bioconductor package *biomaRT*. The CellPhoneDB database was used for LR annotations (Efremova et al., 2020). Multi-subunit LR complexes were not used in this analysis due to the difficulty of annotation. The identified LR pairs by LR hunting were then converted back to their mouse orthologs to create the ranking lists.

The comparison of LR hunting results with those based on STvEA showed that LR hunting was able to detect many STvEA validated LR interactions such as monocyte-derived macrophages and neutrophils (Anxa1-Fpr1 and Anxa1-Fpr2), red-pulp macrophages and neutrophils (Hebp1 and Fpr2), and others (Supplementary Table 1). STvEA integrated CITE-seq and CODEX information to identify the LR pairs, thus the results are reliable. LR hunting method was able to find those validated LR pairs without borrowing the spatially expressed protein information.

### LR Hunting Identified Immune, Epithelial and Stroma Interactions in TNBC

**FIGURE 2:**
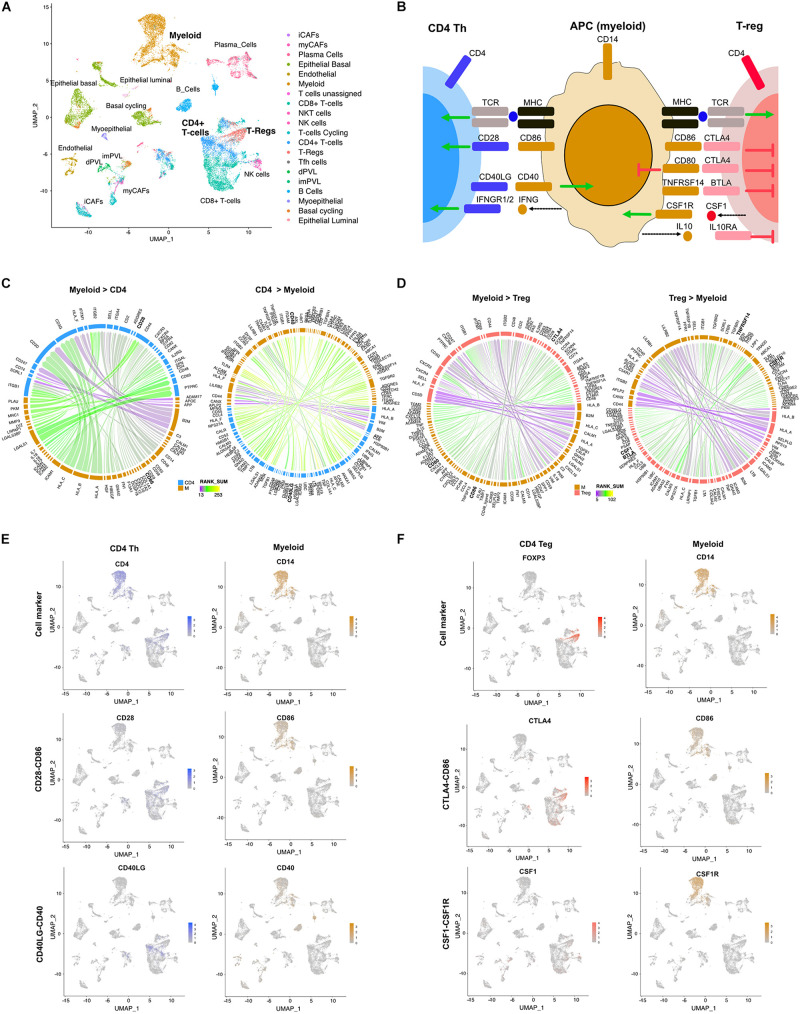
Differential LR interactions between myeloid cells and either Th or Treg cells. **(A)** UMAP plot shows distinct cell populations in five TNBC tumors. **(B)** Image shows known interactions identified by LR hunting between myeloid antigen presenting cells and either CD4 Th or Tregs. Colored arrows indicate direction of signaling from ligand to receptor for stimulatory (green) and inhibitory (red) events. Dashed arrows indicated secreted ligands. Circos plots show the top interactions and by direction for **(C)** myeloid and CD4 T cells and **(D)** myeloid and Treg cells. LR interactions are colored by interaction strength (rank sum) and line thickness represents the frequency of interaction across the five tumors. **(E)** UMAP plots show expression of CD4 Th and myeloid cell markers and expression of LR interactions for CD28-CD86 and CD40L-CD40. **(F)** UMAP plots show expression of Treg and myeloid cell markers and expression of LR interactions for CTLA4-CD86 and CSF1-CSF1R.

Triple-negative breast cancer is a diverse disease with both tumor (Lehmann et al., 2011) and stromal heterogeneity (Wu et al., 2020). Stromal-immune interactions can alter immune cell function (Gruosso et al., 2019). We applied the LR hunting approach to scRNA-seq data from five TNBC tumors to identify LR interactions between myeloid cells and either CD4 T helper (Th) cells or regulatory T cells (Treg) (Figure 2A). Professional antigen-presenting cells (APCs) such as macrophages, B cells and dendritic cells, present foreign antigens loaded on MHC-II to CD4+ Th cells. To fully activate, Th cells require a second interaction between the co-stimulatory CD80/CD86 ligands expressed on APCs and the CD28 receptor on CD4+ T cells (Figure 2B). In addition, CD4+ cells can also be converted to Treg through consumption of IL-2 or other inhibitory cytokines, such as transforming growth factor beta (TGF−β), IL−10, and IL−35. Once converted, Tregs can interact with APCs through the immune checkpoint CTLA−4 interacting with CD80/86, impairing APCs function (Figure 2B). Using our approach, we identified several known interactions between CD4 Th cells and myeloid APC cells, such as the costimulatory CD28-CD86 interaction, the immune activating myeloid secreted interferon gamma (IFNG) with IFNGR1/2 on CD4 cells and CD40LG-CD40 (Figures 2B,C). Furthermore, we were able to identify inhibitory interactions between myeloid and Treg such as CTLA4 on Tregs interacting with either CD80 or CD86, BTLA on Tregs interacting with TNFRS14 on APCs, secreted IL10 binding to the IL10RA on T cells and secreted CSF1 interacting with CSF1R on APC cells (Figures 2B,D). Examination of scRNA expression show that CD4-myeloid cell interactions (CD28-CD86 and CD40LG-CD40) and Treg-myeloid interactions (CTLA4-CD86 and CSF1-CSF1R) are expressed in appropriate cell types (Figures 2E,F).

**FIGURE 3:**
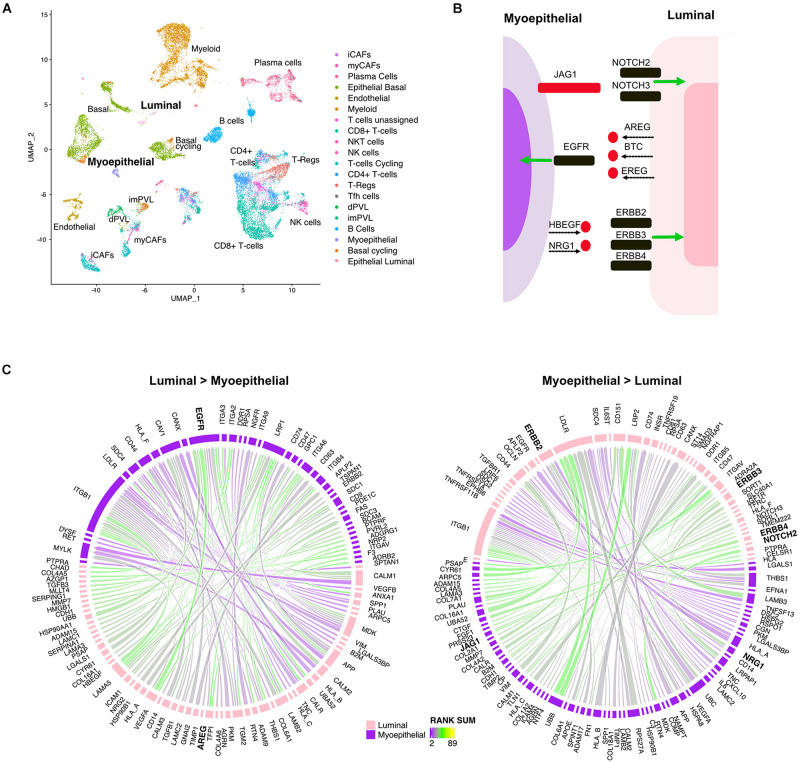
Multiple ligand receptor interactions between luminal epithelial cells and myoepithelial cells. **(A)** UMAP plot shows distinct cell populations in five TNBC tumors. **(B)** Image shows unique ligand (red) receptor (black) interactions between myoepithelial and luminal breast cells. **(C)** Circos plots show the top interactions and by direction between myoepithelial and luminal cells. LR interactions are colored by interaction strength (rank sum) and line thickness represents the frequency of interaction across the five tumors.

Mammary glands consist of two differentiated epithelial cell types organized into an inner layer of luminal epithelial and an outer layer of myoepithelial cells in direct contact with the basement membrane. To better understand the directional signaling events between these two cell types in TNBC, we applied the LR hunting approach to identify interactions between luminal and myoepithelial cells (Figure 3A). We identified distinct directional interactions with multiple epidermal growth factor receptor (EGFR) ligands (AREG, BTC, and EREG) with the EGFR on myoepithelial cells (Figures 3B,C). This signaling is consistent with the previously observed higher expression of EGFR in myoepithelial cells and that overexpression of EGFR can drive cells toward a myoepithelial phenotype in 3D culture (Ingthorsson et al., 2016). We also overserved several ligands (HBEGF and NRG1) interacting with multiple human EGFRs (ERBB2, ERBB3, and ERBB4) expressed on luminal cells (Figures 3B,C). In addition, we identified JAG1 ligand on myoepithelial cells interacting with either NOTCH2 or NOTCH3 on luminal epithelial cells, consistent with others observing NOTCH3 expression in luminal epithelial cells and JAG1 expression in the surrounding myoepithelial layer (Reedijk et al., 2005). Together these interactions describe complex multiple LR interactions that occur between two mammary epithelial cell types.

**FIGURE 4:**
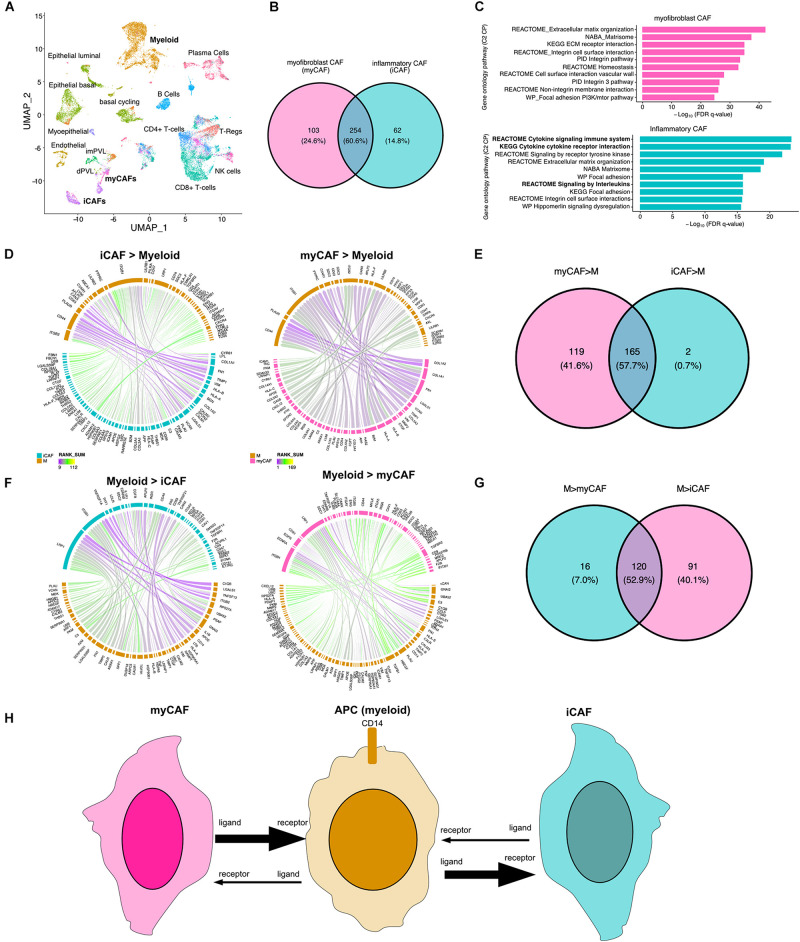
Direction of LR interactions favor myofibroblast CAFs to myeloid and myeloid to inflammatory CAFs. **(A)** UMAP plot shows distinct cell populations in five TNBC tumors with cells of interest in bold. **(B)** Venn diagram of interactions between myeloid and myCAF with interaction between myeloid and iCAF. **(C)** Gene ontology pathway analysis (C2 canonical pathways) of differential interactions unique to myCAFs (pink) or iCAFs (blue). **(D)** Circos plots show the top interactions between iCAF and myeloid cells (iCAF > myeloid) or myCAF to myeloid (myCAF > myeloid). **(E)** Venn diagram shows overlap of interactions between CAFs and myeloid cells. **(F)** Circos plots show the top interactions between myeloid cells and iCAFs (iCAF > myeloid) or myCAFs (myCAF > myeloid). **(G)** Venn diagram shows overlap of interactions between myeloid to CAF interactions. **(H)** Summary of directional interactions between myeloid cells and myCAFs or iCAFs.

Cancer-associated fibroblasts (CAFs) are a major component of the tumor microenvironment and can augment many characteristics of carcinogenesis including extracellular matrix remodeling, angiogenesis, cancer cell proliferation, invasion, and inflammation. Two distinct populations of CAFs have been recently described in scRNA: one with features of myofibroblasts (myCAFs) and the other characterized by high expression of growth factors and immunomodulatory molecules (iCAFs) (Wu et al., 2020). To better understand how myeloid cells interact with CAFs, we applied our LR hunting approach between myeloid and either iCAF or myCAF cells (Figure 4A). We compared the interactions identified between each and show that 60% of the interactions are shared between iCAF and myCAF cells with myeloid cells (Figure 4B). Gene ontology pathway analysis interactions present in myCAFs enriched for extracellular matrix, integrin and focal adhesion (Figure 4C). However, the top pathways enriched in iCAF interactions were immune related (cytokine signaling and signaling by interleukins) in addition to extracellular matrix, focal adhesion and integrin pathways. Further examination of signaling between either iCAF or myCAF to myeloid cells revealed that myCAFs were interacting more as ligands to myeloid cells (Figures 4D,E). However, the opposite was true for myeloid ligands, in which the majority of the interactions occurred between iCAFs (Figures 4F,G). Therefore, myCAFs appear to signal to myeloid cells, whereas myeloid cells provide ligands to iCAFs and the presence or absence of myeloid cells may lead to differential activation of iCAFs (Figure 4H).

For all TNBC cell pairs analyzed above, we also compared our LR hunting method with another well-known method SingleCellSignalR (Cabello-Aguilar et al., 2020). SingleCellSignalR utilizes LR score, which is a penalized LR expression product, to rank the LR pairs. We compared results of CD4-myeloid interactions between our methods with the SingleCellSignalR method. The rankings of results by these two methods were strongly correlated (0.90–0.96) (Supplementary Figure 1). The top 25 interactions agreed well (∼60% were identified by both methods), however 20% of the interactions were identified by only one method. Most of the unique interactions identified by SingleCellSignalR involved B2M and TCR interactions, while the LR hunting method identified additional key interactions (CCL5-CCR1, LGALS1-PTPRC, IFNG-IFNGR2, and CD40LG-CD4), which were not identified by LR score (Supplementary Figure 1). The full ranking lists of TNBC analysis using LR hunting and SingleCellSignalR were listed in Supplementary Tables 2, 3, respectively.

## Discussion

We analyzed scRNA-seq data in a multivariate framework to identify the complex interactions between genes in different cell types and the gene pairs that are most significantly associated with each other. Traditional approaches conduct modeling of each individual LR pair without considering the correlation and high-order interaction patterns in single-cell gene expression data. To analyze the high dimensional scRNA-seq data, we first leveraged information from known LR gene pairs to filter the genes, and then used non-parametric RF approaches which had flexible statistical assumptions for the distribution of gene expression levels and non-linear dependence of gene pairs. The merit of this approach is that after accounting for correlations and interactions multivariately, the discoveries of interacted gene pairs could be more consistent and reproducible. To account for unequal cell type distributions in different samples, we also implemented an approach that computed *p*-values for aggregated IMDI scores based on empirical distributions.

Using our approach, we were able to identify known interactions between differing CD4+ T cells and myeloid cells in TNBC. We also provided evidence that the directional signaling between myCAFs and iCAFs with myeloid cells is not proportional and majority of the interactions occur in the directions from myCAFs to myeloid, and myeloid to iCAFs. One limitation of our study is that only one ligand and one receptor gene pair were analyzed together in our models. Further work is needed to model complex protein structures with multiple receptors functioning as multi-subunit complexes.

## Data Availability Statement

TNBC scRNA-seq data was downloaded from European Nucleotide Archive (ENA) under the accession code PRJEB35405.

## Author Contributions

XSC: conception, design, and study supervision. ML and XSC: development of methodology. XS and YB: data acquisition. ML, YS, TCS, AC, XS, YB, LW, BDL, and XSC: analysis and interpretation. ML, LW, BDL, and XSC: writing, review, and/or revision of the manuscript. All authors contributed to the interpretation of the results, read and approved the manuscript.

## Conflict of Interest

The authors declare that the research was conducted in the absence of any commercial or financial relationships that could be construed as a potential conflict of interest.

## Publisher’s Note

All claims expressed in this article are solely those of the authors and do not necessarily represent those of their affiliated organizations, or those of the publisher, the editors and the reviewers. Any product that may be evaluated in this article, or claim that may be made by its manufacturer, is not guaranteed or endorsed by the publisher.
