# Supplementary material for: LR Hunting: A Random Forest Based Cell–Cell Interaction Discovery Method for Single-Cell Gene Expression Data
Source: Front Genet. 2021 Aug 20;12:708835. doi: 10.3389/fgene.2021.708835 (PMC8420858; doi:10.3389/fgene.2021.708835)

Figure S1. Comparison of LR score and LR hunting interactions for the analysis of TNBC dataset. Scatterplots showing the rankings of top 25 interactions identified by LR score and LR hunting methods with estimated Spearman correlations for A) tumor 1, B) tumor 2, C) tumor 3 and D) tumor 4. Data points are colored by the absolute values of the differences in ranks between the methods(Δ rank). Venn diagrams show the overlap between the top 25 interactions identified by both methods and the interactions unique to each method.

Figure S1

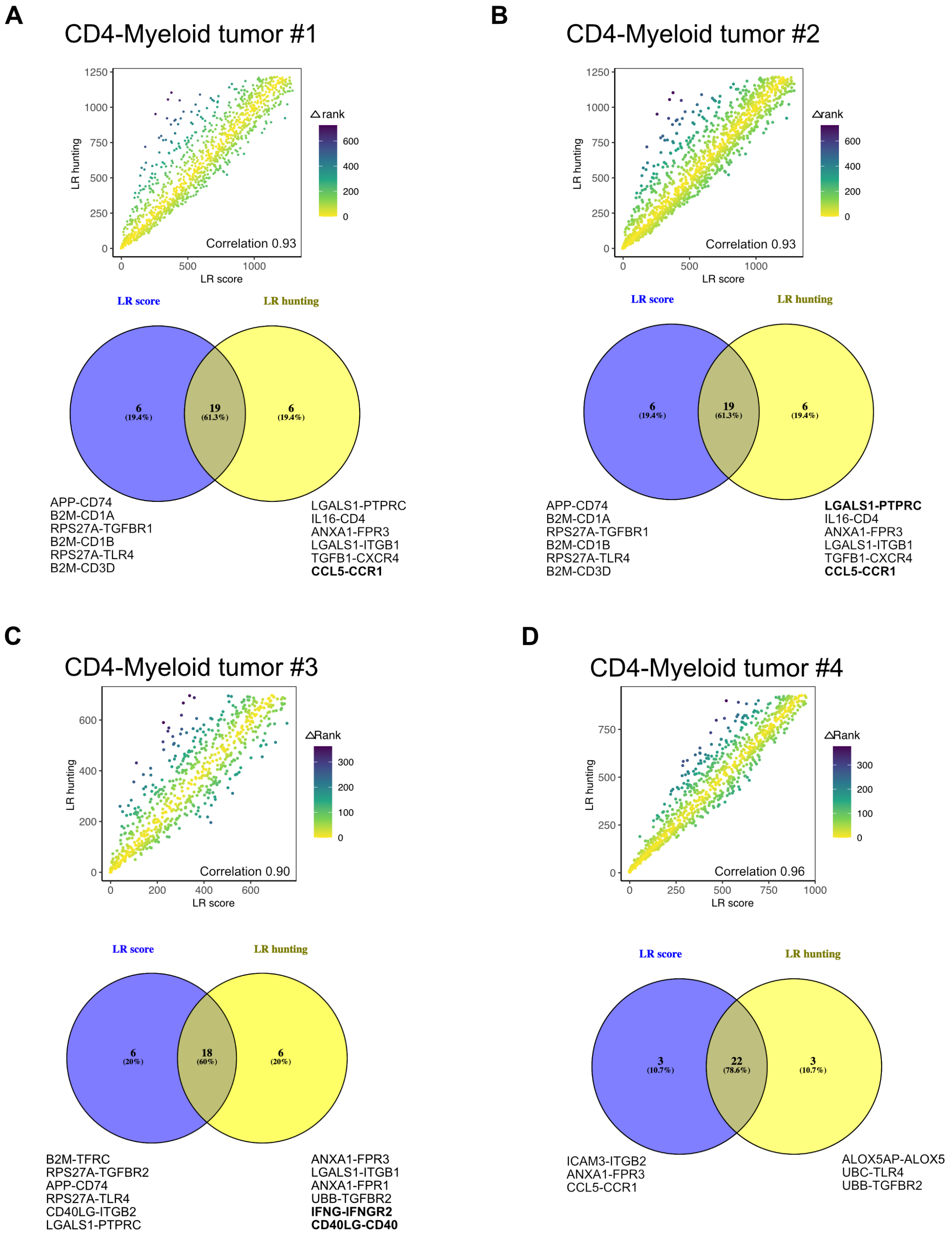

Supplement: Supplementary file 1 [file Data_Sheet_1.PDF]
